# A Machine Learning Based Causal Interface for Time Varying Environmental Predictors of Substance Use Initiation in the ABCD Study

**DOI:** 10.64898/2026.04.15.26350988

**Published:** 2026-04-17

**Authors:** Mengman Wei, Lasya Yadlapati, Qian Peng

**Affiliations:** 1 Department of Neuroscience, The Scripps Research Institute, 10550 N Torrey Pines Rd, La Jolla, 92037, CA, U.S.; 2 University of California, San Diego, 9500 Gilman Dr, La Jolla, CA, 92093, USA

**Keywords:** substance use initiation, time-varying environmental factors, longitudinal analysis, adolescence, ABCD Study

## Abstract

**Background:**

The Adolescent Brain Cognitive Development (ABCD) Study^®^ offers rich longitudinal data on environmental, genetic, and other factors related to substance use initiation. Classical marginal structural models (MSMs) require selecting covariates for propensity models, which is challenging in the presence of hundreds of correlated predictors.

**Methods:**

We analyzed longitudinal panel data from 11,868 ABCD participants, where each individual contributed repeated observations over time. Interval-level binary outcomes were defined for initiation of alcohol, nicotine, cannabis, and any substance, restricting analyses to participants at risk prior to initiation. All predictors were constructed as lagged variables to preserve temporal ordering. We implemented a two-stage machine learning–based causal framework. First, we performed graph discovery using a Granger-inspired lagged predictive modeling approach, applying elastic-net logistic regression to identify predictive relationships between lagged environmental variables and future initiation outcomes. Robust candidate edges were selected using subject-level bootstrap stability selection. Second, we estimated adjusted effect sizes for stable edges using double machine learning (DML)–style partialling-out with cross-fitting. For each candidate predictor, the treatment was defined as the lagged variable of interest and adjusted for high-dimensional lagged covariates. Cross-fitting with group-based splitting accounted for within-subject dependence, and nuisance functions were estimated using random forest models. Cluster-robust standard errors were used for inference.

**Results:**

We identified a set of stable predictors across multiple domains, including sleep patterns, family environment, peer relationships, behavioral traits, and genetic risk. Many predictors were shared across substance outcomes, while some were outcome-specific. Estimated effect sizes were modest, typically ranging from −0.01 to 0.02 per standard deviation increase in the predictor. Both risk-increasing and protective associations were observed. Risk factors included sleep disturbance and behavioral risk indicators, while protective factors included parental monitoring and structured environments.

**Conclusions:**

This study provides a practical framework for analyzing high-dimensional longitudinal data and identifying time-varying predictors of substance use initiation. The approach combines machine learning for variable selection with causal inference methods for effect estimation. The results highlight both shared and substance-specific risk factors and identify modifiable targets, such as family environment and sleep, that may inform prevention strategies.

## Introduction

Substance use often begins during adolescence, a developmental period when curiosity, risk-taking, and social influences increase while the brain is still maturing. Initiation of alcohol, nicotine, or cannabis use at earlier ages is consistently associated with a higher risk of later substance use disorders and other adverse outcomes, making prevention and early identification of risk a major public health priority [1].

Risk for initiation is multifactorial and spans multiple domains, including individual behavior and mental health, family environment and parenting, peer context, school experiences, and neighborhood and socioeconomic conditions. These influences can change over time, and their timing may matter. For example, changes in peer exposure or parental monitoring during a specific developmental window may be more predictive of near-term initiation than baseline-only measures. Large longitudinal cohorts therefore provide an opportunity to study both whether initiation occurs and when it occurs, using modeling frameworks that respect time ordering [2, 3].

The Adolescent Brain Cognitive Development (ABCD) Study^®^ is particularly well suited for this work because it is a large, diverse, multi-site longitudinal cohort that follows youth from late childhood into adolescence with rich repeated assessments across psychosocial, environmental, and health domains [4, 5].

Methodologically, two complementary goals are important. First, we need a principled way to screen thousands of time-varying predictors to identify candidate directional relationships that respect temporal precedence (past → future). A common approach is Granger-style analysis, where a predictor is considered informative if its past values improve the prediction of a later outcome, with directionality arising from time ordering rather than simultaneity [6, 7]. In high-dimensional settings, sparse models such as elastic-net logistic regression can perform variable selection while handling correlated predictors, and stability selection (repeated resampling or subsampling) can help distinguish robust signals from chance selections [8, 9, 10, 11, 12]. Second, after identifying robust candidate edges, we want effect-size estimates that adjust for confounding from other past variables. Double/debiased machine learning (DML) provides a framework for estimating low-dimensional effects while flexibly modeling high-dimensional nuisance relationships with machine learning and using cross-fitting to reduce overfitting bias [13, 14, 15, 16].

**Figure 1:**
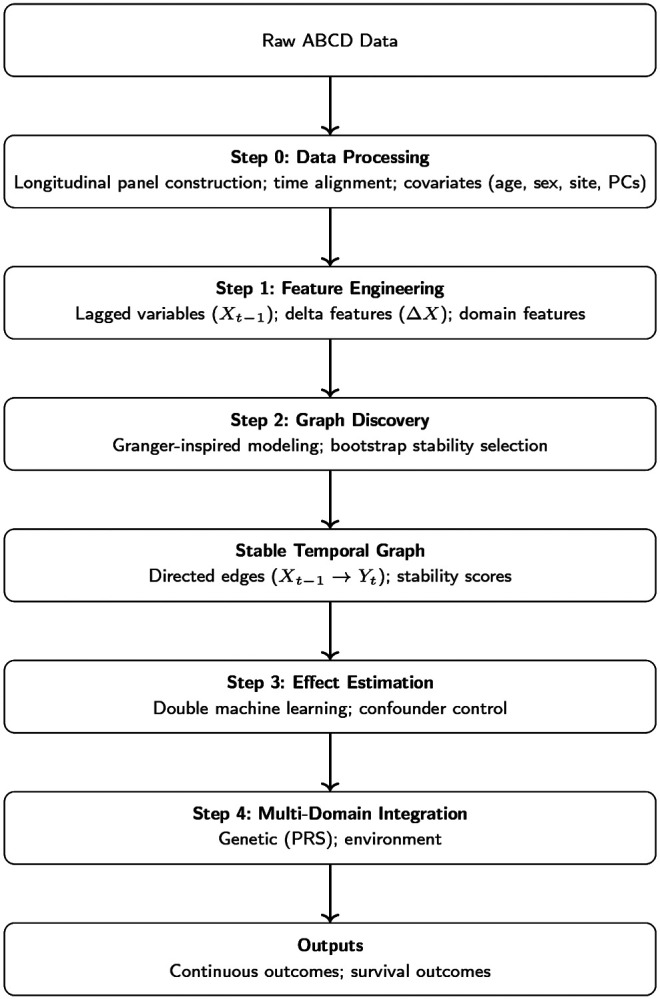
Overview of the multi-stage modeling pipeline. This study uses a structured pipeline to integrate longitudinal, genetic, and environmental data from the ABCD Study. First, raw data are processed into a time-aligned longitudinal panel with covariate adjustment. Next, feature engineering constructs lagged and change (delta) variables to capture temporal dynamics. A graph discovery step then identifies stable temporal relationships using Granger-inspired lagged predictive modeling with bootstrap stability selection. Based on the resulting temporal graph, causal effects are estimated using double machine learning while controlling for confounders. Finally, features from multiple domains, including polygenic risk scores, and environmental exposures are integrated to model both continuous behavioral outcomes and time-to-event outcomes related to substance use initiation.

In this work, we combine these ideas into a two-stage pipeline: (i) graph discovery using lagged predictors, sparse logistic regression, and subject-level bootstrap stability to propose directional edges; and (ii) effect estimation for stable edges using DML-style partialling-out with cross-fitting, with machine-learning nuisance models and cluster-robust uncertainty to account for repeated measures within individuals (Figure 1). Together, this approach aims to provide an interpretable causal interface that highlights which time-ordered factors are most consistently linked to subsequent initiation risk, and estimates their adjusted associations under standard causal assumptions.

## Methods

### Cohort

We used data from the Adolescent Brain Cognitive Development (ABCD) Study^®^ release 5.1 [5]. The analytic sample included participants with: (i) genome-wide genotyping data passing quality control; (ii) at least one visit with substance use data; and (iii) available data for core covariates (sex, age, site, and ancestry principal components). From the full ABCD baseline cohort (*N* = 11,869), the analytic sample comprised *N* = 11,868 children after applying these criteria.

In the analytic cohort (*N* = 11,868; mean baseline age 9.91 ± 0.62 years), substance-use initiation was observed during follow-up for alcohol (4,330/11,868; 36.5%), nicotine (646/11,868; 5.44%), cannabis (406/11,868; 3.42%), and any substance (4,706/11,868; 39.7%).

Among individuals who initiated use, the estimated mean (SD) age at initiation was 20.83 (1.82) months from baseline year age for alcohol (median 20.75; IQR 19.42–21.83), 22.65 (1.93) for nicotine (median 22.83; IQR 21.33–24.17), 23.30 (1.70) for cannabis (median 23.42; IQR 22.19–24.65), and 20.93 (1.85) for any substance (median 20.92; IQR 19.50–21.92).

Sex differences were modest. Event rates were slightly higher in males than in females for alcohol (39.6% vs. 36.7%) and any substance (43.1% vs. 39.7%). In contrast, nicotine initiation rates were similar between males and females (5.29% vs. 6.02%), as were cannabis initiation rates (3.76% vs. 3.51%). Participants with unknown sex showed consistently lower initiation rates across all outcomes (alcohol 26.6%, nicotine 4.36%, cannabis 2.18%, any substance 29.4%).

Substantial differences were observed across race/ethnicity groups. Alcohol initiation rates were highest in White participants (42.7%, *n* = 6,173), followed by Other (37.3%, *n* = 1,248), Hispanic (32.6%, *n* = 2,410), Asian (33.3%, *n* = 252), and Black participants (20.1%, *n* = 1,784). A similar pattern was observed for any-substance initiation, with rates of 44.8% in White, 40.9% in Other, 36.9% in Hispanic, 34.1% in Asian, and 25.3% in Black participants. Nicotine and cannabis initiation rates were generally lowest among Asian participants (0.79% and 0.40%, respectively). Additional details are provided in our previous work [17].

### Study design and data format (longitudinal panel data)

We analyzed longitudinal data in which each participant contributes multiple observations over time, following a standard panel data framework [18, 19]. Each row in the dataset corresponds to one discrete follow-up interval (indexed by step).

For each substance outcome (e.g., alcohol, nicotine, cannabis), we defined an interval-level binary outcome indicating whether initiation occurred during that interval (1 = initiated in that interval, 0 = did not initiate in that interval). Because participants are only at risk of initiation prior to first use, we additionally defined an at-risk (validity) indicator m_*_ for each outcome. Specifically, m_*_ = 1 indicates that the participant is still at risk and the corresponding row is included for that outcome, whereas m_*_ = 0 indicates that the row is excluded for that outcome.

To ensure proper temporal ordering and avoid information leakage, all candidate predictors were constructed using lagged values from prior intervals [18]. Lagged variables were denoted using the suffix _L*k*, where *k* represents the lag length (e.g., _L1 corresponds to the previous interval and _L2 to two intervals earlier). The analysis focused exclusively on these lagged predictors so that all predictors temporally precede the outcomes.

### Step 1: Graph Discovery Using Lagged Prediction and Bootstrap Stability

We first identified candidate directional relationships from past predictors to future outcomes using a Granger-inspired lagged predictive modeling approach, in which a predictor is considered potentially influential if its past values improve the prediction of a future outcome [7].

#### Eligibility criteria.

For each outcome, we included only rows that satisfied two conditions: (1) the participant was at risk for that outcome, and (2) at least one prior interval was available. To ensure stable estimation, we further excluded targets with fewer than 500 eligible rows, extremely low or extremely high prevalence, or no variation in the outcome.

#### Candidate predictors.

The predictor set consisted of all lagged variables with suffixes _L1 through _Lmax_lag. Identifier and bookkeeping fields were excluded. Additional details are provided in our previous work [17].

#### Preprocessing.

All predictors were converted to numeric format. Infinite values were treated as missing. Missing values were imputed using the column median, with a fallback value of 0 when necessary. Predictors were then standardized (z-scored) to ensure comparability across variables.

#### Sparse predictive model.

For each outcome, we fit an elastic-net logistic regression model to predict the interval-level outcome from lagged predictors. Elastic-net regularization promotes sparsity and handles correlated predictors effectively [12]. Class weighting was applied to account for class imbalance.

#### Bootstrap stability selection.

To assess robustness, we repeated model fitting across bootstrap samples drawn at the subject level. For each bootstrap replicate, we recorded predictors with non-zero coefficients. For each predictor–outcome pair, we computed a stability score defined as the proportion of bootstrap samples in which the predictor was selected. This procedure follows the principles of stability selection for robust variable identification [11].

#### Outputs of discovery.

This step produced a set of candidate directed edges (predictor → outcome) with associated bootstrap stability scores, along with ranked coefficient summaries from the fitted models.

### Step 2: Effect estimation for stable edges using DML-style partialling-out with cross-fitting

After identifying candidate edges, we estimated adjusted effect sizes using DML-style partialling-out with cross-fitting, a framework for adjusted (and causal under assumptions) estimation with machine learning–based nuisance models [20].

#### Stable edge selection.

We retained edges exceeding a predefined stability threshold (e.g., ≥ 0.6) and limited the number of edges per outcome.

#### Estimation dataset.

Effect estimation was conducted using training and validation splits, excluding the test set. For each outcome, only rows with m_*_ = 1 and step ≥ 1 were included.

#### Treatment, outcome, and covariates.

- Outcome: interval-level outcome (y_*_)
- Treatment: lagged predictor
- Covariates: all lagged variables up to the same lag order (excluding the treatment)

All covariates were numerically encoded, imputed, and standardized.

#### Cross-fitting.

To account for repeated measures within individuals, we used group-based cross-validation (GroupKFold) splitting by participant ID. Cross-fitting reduces overfitting bias in causal estimation [20].

#### Nuisance models.

We estimated:

- Outcome model: $\hat{m}(X)=E[Y\;|\;X]$
- Treatment model: $\hat{g}(X)=E[D\;|\;X]$

using random forest regression [21].

Residuals were computed as:

(1) $$\tilde{Y}=Y-\hat{m}(X)$$

(2) $$\tilde{D}=D-\hat{g}(X)$$

#### Effect estimation.

The adjusted effect was estimated by regressing the residualized outcome on the residualized treatment:

$$\tilde{Y}=Y-\hat{m}(X),\;\;\tilde{D}=D-\hat{g}(X),$$

$$\tilde{Y}=\theta \tilde{D}+\varepsilon .$$

The coefficient θ represents the adjusted effect on the probability scale.

#### Uncertainty estimation.

Because observations are clustered within individuals, we computed cluster-robust standard errors using a sandwich estimator [22].

#### Output.

This step produced effect estimates, standard errors, and confidence intervals for each retained edge.

### Graph Discovery Summary

The graph discovery step identifies temporally ordered predictive relationships using lagged variables and bootstrap stability. While this step suggests directional associations, it does not establish causality. The DML step provides adjusted effect estimates under standard causal assumptions, including correct temporal ordering and adequate control of confounding through lagged covariates. Together, these steps could yield a robust and interpretable framework for identifying and quantifying candidate risk pathways.

### Sample Characteristics

## Results

### Graph discovery and stability selection

#### Overview of stability patterns

The graph discovery step identified a large number of candidate lagged predictors across all outcomes. After bootstrap stability selection, many predictors showed high stability (≥ 0.80), indicating that they were consistently selected across resampled datasets. Full details of these results are provided in the Supplementary Material.

**Figure 2:**
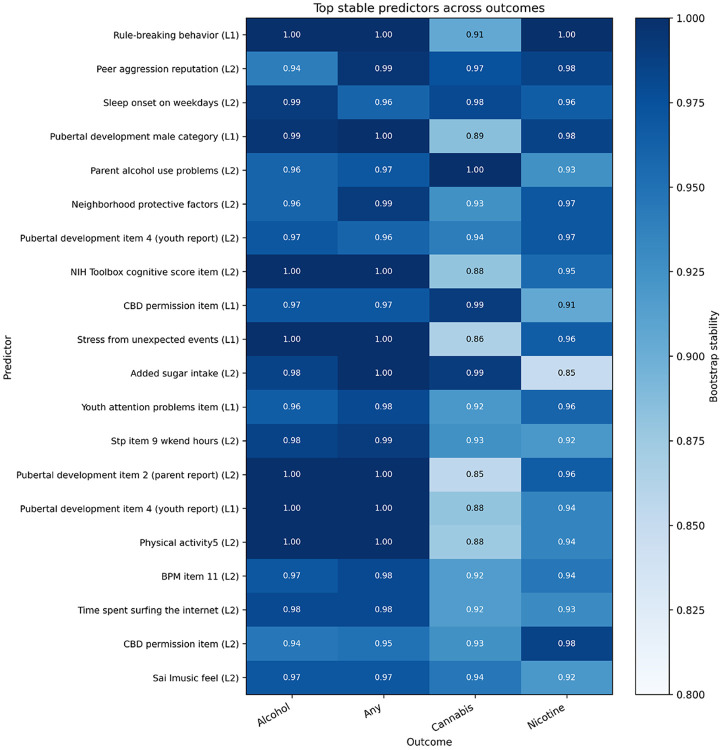
Heatmap of top stable predictors across outcomes. Each cell shows the bootstrap stability score (proportion of times selected across resamples) for a predictor–outcome pair. Higher values indicate more consistent selection. Predictors appearing across multiple outcomes suggest shared risk pathways.

Across outcomes, several predictors showed high stability simultaneously, as shown in Figure 2. These included variables related to:

- sleep and circadian rhythm,
- parental and family environment,
- peer relationships,
- pubertal development,
- prior substance-related exposure.

This pattern suggests that a shared set of upstream factors contributes to multiple substance use initiation outcomes. Cannabis showed slightly lower stability for some predictors compared to alcohol and any-substance outcomes, indicating greater variability in predictor selection for this outcome.

#### Top predictors by outcome

**Figure 3:**
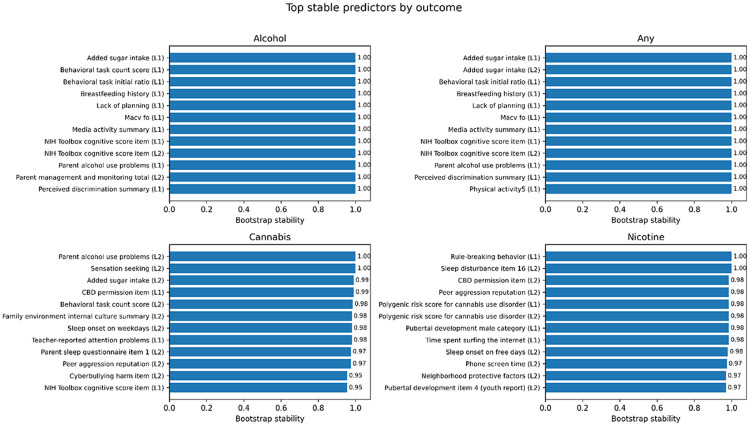
Top stable predictors by outcome. For each substance use outcome, predictors are ranked by bootstrap stability. Only the most stable predictors are shown. This figure highlights both shared and outcome-specific predictors.

When examining predictors within each outcome, both shared and outcome-specific patterns were observed, as shown in Figure 3. Full details of these results are provided in the Supplementary Material.

For alcohol and any-substance initiation, the top predictors spanned multiple domains, including polygenic risk scores (PRS), parental substance use and family environment, early-life factors (e.g., breastfeeding), and behavioral and health-related measures.

For cannabis initiation, predictors were more concentrated in behavioral traits (e.g., sensation seeking), family and parenting factors, and sleep and circadian measures.

For nicotine initiation, the top predictors included polygenic risk scores (PRS) for substance use, sleep disturbance, screen time and behavioral factors, and pubertal development.

Overall, these results indicate that substance use initiation is influenced by a combination of shared and outcome-specific factors.

### DML-based effect estimation

#### Global effect estimates

We next estimated adjusted effects for stable predictors using a double machine learning (DML) partialling-out approach with cross-fitting.

**Figure 4:**
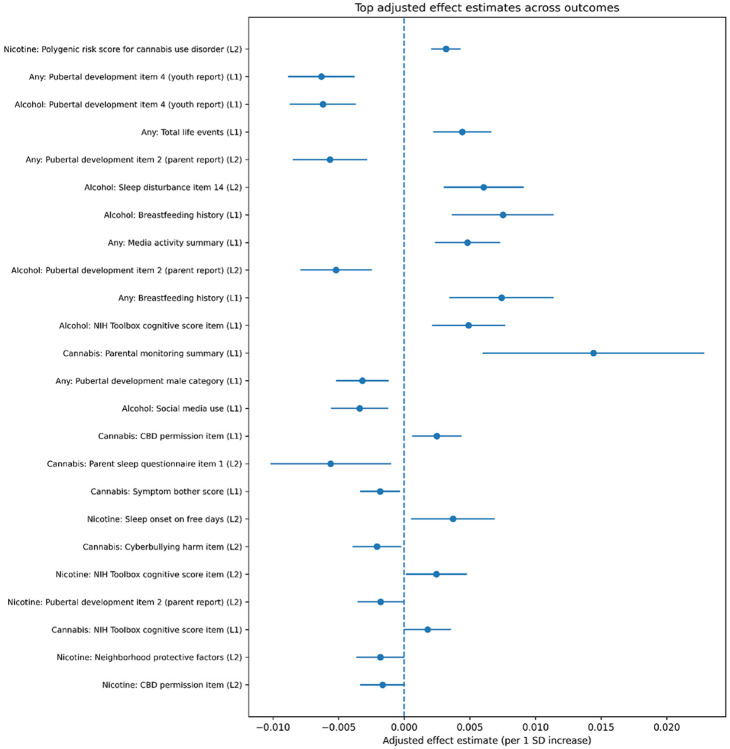
Top DML-style effect estimates across all outcomes. Points represent estimated effects per 1 standard deviation increase in the predictor, and horizontal lines show 95% confidence intervals. The dashed vertical line indicates zero effect.

Across all outcomes, effect sizes were modest in magnitude. Most estimates ranged between approximately −0.01 and 0.02 per 1 standard deviation increase in the predictor, as shown in Figure 4. Full details of these results are provided in the Supplementary Material.

Both positive and negative associations were observed:

- Positive effects (increased risk) were observed for factors such as genetic risk, sleep disturbance, and behavioral risk indicators.
- Negative effects (protective associations) were observed for factors related to parental monitoring and structured environments.

Some confidence intervals included zero, reflecting uncertainty in estimation in a high-dimensional longitudinal setting.

#### Outcome-specific effects

**Figure 5:**
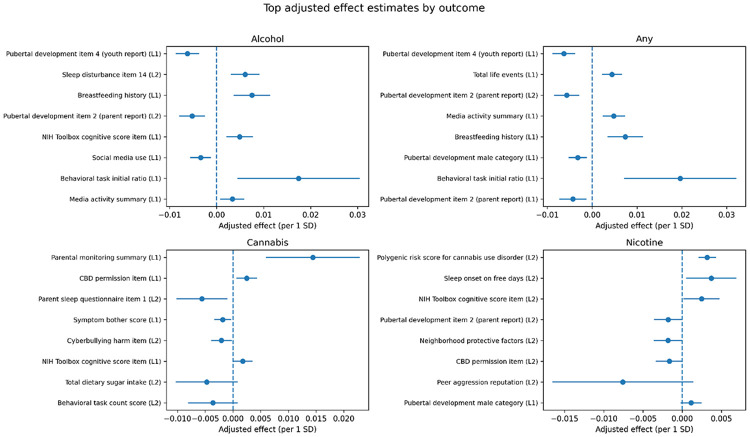
Top DML-style effect estimates by outcome. For each outcome, the most important predictors are shown with estimated effects and 95% confidence intervals. This figure highlights differences in effect patterns across substances.

More detailed patterns were observed when examining effects by outcome (Figure 5). Full results are provided in the Supplementary Material.

For alcohol initiation, increased risk was associated with sleep disturbance, screen time, and behavioral factors.

For any-substance initiation, increased risk was associated with life stress, total adverse events, and reduced parental structure.

For cannabis initiation, the strongest effect was observed for parental monitoring, which showed a protective association. Additional predictors included behavioral and environmental exposures.

For nicotine initiation, increased risk was associated with genetic liability (PRS), sleep and circadian measures, and peer-related behavioral factors.

These findings indicate that, while many predictors are shared, each substance shows a distinct profile of associated factors.

#### Summary of key findings

Across both the graph discovery and effect estimation steps, three main patterns emerged:

1. Many predictors showed high stability, indicating robust temporal associations.
2. A shared set of environmental and behavioral factors contributed to multiple substance outcomes.
3. Effect sizes were modest but consistent in direction, with both risk-increasing and protective associations identified.

## Discussion

### Main findings

In this study, we developed and applied a machine learning–based causal framework to identify and quantify time-varying predictors of substance use initiation in a large longitudinal cohort. We found that a broad set of predictors across multiple domains, including genetic, behavioral, family, and environmental factors, were associated with substance use initiation. Many of these predictors were shared across different substances, while others were more specific to individual outcomes. The combination of stability selection and double machine learning (DML)–based estimation allowed us to identify predictors that were both robust and interpretable.

### Shared and substance-specific pathways

A key finding is the presence of both shared and outcome-specific predictors. Shared predictors, such as sleep patterns, family environment, and peer-related factors, suggest that there are common pathways underlying general liability to substance use. These factors may reflect broader behavioral or developmental processes that increase vulnerability across substances.

At the same time, substance-specific patterns were observed. For example, cannabis initiation was more strongly linked to behavioral traits and parental monitoring, while nicotine initiation showed stronger associations with genetic liability and sleep disturbance. These differences highlight the importance of considering both general and specific mechanisms when studying substance use.

### Interpretation of effect sizes

The estimated effects were modest in magnitude. This is expected in population-level longitudinal data, where individual predictors typically explain only a small portion of the total risk. Despite their small size, these effects are meaningful when considered together. Substance use initiation is a complex outcome influenced by many factors, and risk is likely distributed across multiple domains rather than driven by a single strong predictor.

### Modifiable risk factors

Several identified predictors represent potentially modifiable factors, including parental monitoring, sleep and circadian patterns, and screen time and behavioral regulation. These findings suggest that interventions targeting family environment and daily behaviors may help reduce the risk of substance use initiation.

### Methodological contribution

This study also contributes methodologically by providing a scalable framework for analyzing high-dimensional longitudinal data. The two-stage approach combines lagged predictive modeling for temporal ordering, stability selection for robustness, and DML-based estimation for adjusted effects. This framework allows researchers to move from large sets of candidate predictors to a smaller set of interpretable associations while accounting for confounding and repeated measures.

### Limitations

Several limitations should be considered. First, causal interpretation relies on standard assumptions, including correct temporal ordering and adequate control of confounding. Unmeasured confounding may still be present. Second, effect sizes were estimated in a high-dimensional setting, which may introduce variability and uncertainty. Third, measurement error in self-reported variables may affect the results. Finally, the findings are based on the ABCD cohort and may not fully generalize to other populations.

### Future directions

Future work could integrate neuroimaging features into the same framework, examine interactions between genetic and environmental factors, and extend the approach to other behavioral and mental health outcomes.

## Conclusion

In this study, we developed a machine learning–based causal framework to analyze time-varying predictors of substance use initiation in a large longitudinal cohort. We identified a set of robust predictors across multiple domains, including genetic, behavioral, and environmental factors. Many predictors were shared across substances, while others showed outcome-specific patterns. Effect sizes were modest but consistent, and several modifiable factors were identified, including parental monitoring and sleep-related variables. Overall, this approach provides a practical and interpretable way to study complex longitudinal data and to identify potential targets for prevention.

## Data Availability

The analysis code and scripts used in this study are publicly available at: https://github.com/mw742/ABCD-CausalML This study uses data from the Adolescent Brain Cognitive Development (ABCD) Study (https://abcdstudy.org), available through the NIMH Data Archive (NDA). The ABCD data release used was version 5.1. The study is supported by the National Institutes of Health (NIH) and additional federal partners under multiple award numbers, including U01DA041048 and U01DA050987. A full list of funders is available at https://abcdstudy.org/federal-partners.html.
